# Design, synthesis and docking studies of novel thiazole derivatives incorporating pyridine moiety and assessment as antimicrobial agents

**DOI:** 10.1038/s41598-021-86424-7

**Published:** 2021-04-12

**Authors:** Rizk E. Khidre, Ibrahim Ali M. Radini

**Affiliations:** 1 Chemistry Department, Faculty of Science, Jazan University, Jazan, Saudi Arabia; 2 Chemical Industries Division, National Research Centre, Dokki, Giza 12622 Egypt

**Keywords:** Chemical biology, Drug discovery, Chemistry

## Abstract

A novel series of substituted 4,6-dimethyl-2-oxo-1-(thiazol-2-ylamino)-1,2-dihydropyridine-3-carbonitrile derivatives **6**, **9**, **13**, **15**, and **17** was synthesized in a good to excellent yield from the reaction of 1-(3-cyano-4,6-dimethyl-2-oxopyridin-1(2*H*)-yl)thiourea with 2-oxo-*N*'-arylpropanehydrazonoyl chloride, chloroacetone, *α*-bromoketones, ethyl chloroacetate, and 2,3-dichloroquinoxaline, respectively. The potential DNA gyrase inhibitory activity was examined using in silico molecular docking simulation. The novel thiazoles exhibit dock score values between − 6.4 and − 9.2 kcal/mol and they were screened for their antimicrobial activities. Compound **13a** shown good antibacterial activities with MIC ranged from 93.7–46.9 μg/mL, in addition, it shown good antifungal activities with MIC ranged from 7.8 and 5.8 μg/mL.

## Introduction

**Figure 1:**
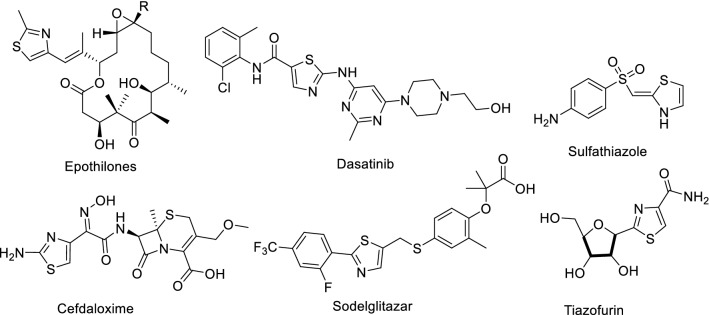
Commercial drugs contain thiazole moiety.

Thiazoles are present in numerous natural products e.g. epithilone, thiostrepton, thiamine pyrophosphate (TPP), carboxylase vitamin B1, and penicillin^1^. Thiazoles have diverse applications in drug development for treatment allergies^2^, inflammation^3^, HIV infections^4^, hypertension^5^, bacterial infections^6^, hypnotics^7^, schizophrenia^8^, and pain^9^, as novel inhibitors of bacterial DNA gyrase B^10^, and as fibrinogen receptor antagonists with antithrombotic activity^11^. They exhibited fabulous pharmaceutical activities for instance antifungal^12^, antimicrobial^13–15^, anti-inflammatory^16,17^, analgesic^18^, and anti-cancer^19,20^, anticonvulsant activities^21^. There are several commercial drugs contain thiazole moiety (Fig. 1).

**Figure 2:**
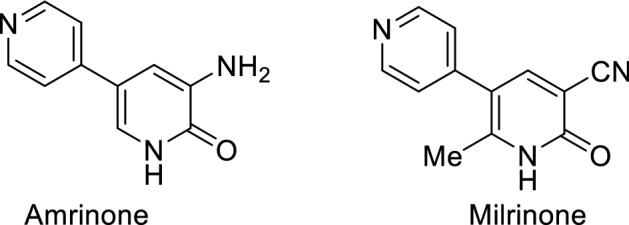
Structure of amrinone and milrinone.

Pyridines are an important class of heterocyclic compounds because they occur in many natural compounds that have biological activity such as vitamin B3 (niacin) and vitamin B6 (pyridoxin) and natural alkaloids^22^. Multi substituted pyridines are significant synthons in heterocyclic synthesis^23–26^. 2-Pyridone derivative appeared as the backbone in over 7,000 drugs^27,28^ for instance amrinone^29^ and milrinone^30^ (Fig. 2) used for treating congestive heart failure (Fig. 2). Compounds containing the pyridine pattern have a wide range of biological profiles including antimicrobial^31–36^, anti-viral^37,38^, antioxidant^39^, antidiabetic^40^, anticancer^41–43^, anti-inflammatory agents^44,45^. For all these benefits related to thiazole and pyridine derivatives and following our work^46–50^, we report here the synthesis of a new library of thiazole derivatives from 1-(3-cyano-4,6-dimethyl-2-oxopyridin-1(2*H*)-yl)thiourea **2**.

## Results and discussion

**Scheme 1:**
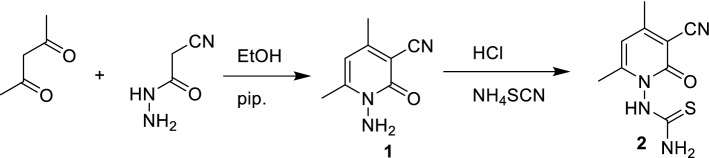
Synthesis of 1-(3-cyano-4,6-dimethyl-2-oxopyridin-1(2*H*)-yl)thiourea **2**.

The precursor *N*-aminopyridone **1** was synthesized from the reaction of acetylacetone with cyanoacetohydrazide in EtOH containing piperidine at reflux temperature^36^. Solution of **1** in conc. HCl was treated with ammonium isothiocyanate then the mixture was heated at reflux temperature to afford white precipitate in excellent yield and identified as 2-oxopyridinyl thiourea **2** based on elemental analyses and spectral data. IR spectrum of **2** showed absorption bands at 3408, 3261, 3219, 2222, 1662 cm^−1^ owing to NH, NH_2_, CN, CO, respectively. ^1^H NMR spectrum revealed two singlet signals at δ 2.20 and 2.27 ppm owing to 2CH_3_, one singlet signal at δ 6.32 ppm due to pyridine-H_5_, two exchangeable signals at δ 7.76 and 10.16 ppm due to NH_2_ and NH, respectively. Its ^13^C NMR spectrum displayed the presence 9 carbon peaks. The most important peaks resonate at δ 159.9 (C=O), 185.5 (C=S). Mass spectrum displayed [M^+^ + 1] ion peak at *m/z* 223.6 (Scheme 1).

**Scheme 2:**
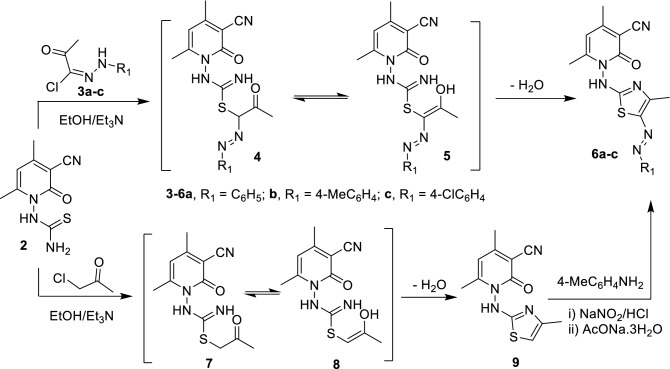
Synthesis of thiazole derivatives **6** and **9**.

**Scheme 3:**
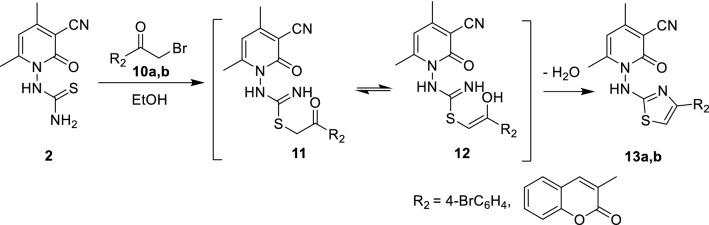
Synthesis of thiazole derivatives **13a,b**.

**Scheme 4:**
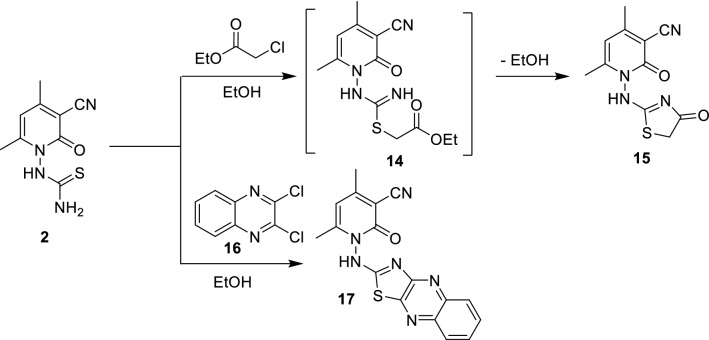
Synthesis of thiazole derivatives **15** and **17**.

The reactivity of thiourea moiety was tested by the reaction of 2-oxopyridinyl thiourea **2** with different reagents as depicted in Schemes 2, 3, 4. Treatment of compound **2** with hydrazonyl chloride **3a-c** in absolute EtOH containing 5 drops of Et_3_N at reflux temperature to afford the corresponding substituted 1,3-thiazole derivatives **6a-c**, in good yields, via nucleophilic substitution followed by cyclization. On the other hand, 4,6-dimethyl-1-((4-methyl-5-(*p*-tolyldiazenyl)thiazol-2-yl)amino)-2-oxo-1,2-dihydropyridine-3-carbonitrile **6b** was prepared by anther rout from the reaction of **2** with chloroacetone to afford 1-(2-thiazolylamino)-2-pyridone **9**, in a high yield, followed by diazotization using 4-methylbenzenediazonium chloride (Scheme 2).

The structure of the compounds **6a-c** and **9** was confirmed. The IR spectrum of compound **6b**, as a representative example, exhibited the lack of NH_2_ and C=S peak at 3261, 3219, and 1269 cm^−1^. The ^1^H-NMR spectrum of **6b** showed new singlet signals at δ 2.37, 2.49 ppm assigned to two methyl, additionally, two doublet signals at δ 7.20 and 7.55 ppm attributable to 4-methylbenzene. Its ^13^C-NMR spectrum revealed the lack of C=S signal at 185.5 ppm and appearance 17 carbon signals. Moreover, the mass spectra of **6b** revealed [M^+^-15] ion peak at *m/z* 383. This clearly indicates the thioamide moiety was involved in cyclization reaction with hydrazonyl chlorides **3a–c** to give 1,3-thiazole derivatives **6a-c**.

Similarly, treatment compound **2** with an equimolar amount of *α*-bromoketones, 2-bromo-1-(4-bromophenyl)ethan-1-one **10a** and 3-(2-bromoacetyl)-2*H*-chromen-2-one **10b**, in ethanol at reflux temperature afforded 4,6-dimethyl-1-((4-substitutedthiazol-2-yl)amino)-2-oxo-1,2-dihydropyridine-3-carbonitrile **13a**,**b**; respectively (Scheme 3). ^1^H NMR spectrum of **13a** showed singlet signal at δ 7.53 ppm owing to thiazole-H_5_, in addition, two doublet of doublets signals at δ 7.56 and 7.67 ppm (*J* = 2 Hz, 9 Hz) due to 4-bromobenzene. Its ^13^C-NMR spectrum revealed the lack of C=S signal and appearance 15 carbon signals. Its mass spectrum revealed 401 [M^+^ + 1] (100%).

Next, thiourea derivative **2** was reacted with ethyl chloroacetate and 2,3-dichloroquinoxaline **16** in ethanol at reflux temperature to yield 4,6-dimethyl-2-oxo-1-((4-oxo-4,5-dihydrothiazol-2-yl)amino)-1,2-dihydropyridine-3-carbonitrile **15** and 4,6-dimethyl-2-oxo-1-(thiazolo[4,5-*b*]quinoxalin-2-ylamino)-1,2-dihydropyridine-3-carbonitrile **17**, respectively, in good yields (Scheme 4). The IR spectra of **15** exhibited new strong band corresponding to C=O at 1737 cm^−1^ and disappearance thioamide moiety. The ^1^H NMR spectra exhibited new singlet for methylene in thiazole ring at δ 4 ppm. Its ^13^C NMR spectra showed 11 carbon peaks e.g., CH_2_ and CO in thiazole ring exhibited at δ 34.4 and 173.7 ppm, respectively. Its mass spectrum displayed [M^+^] peak at 262 (90%).

### Molecular docking studies and antimicrobial activity

The innovative arylthioureas were docked to the active site of DNA gyrase enzyme using Autodock 4. We studied the hypothetical binding approach of 9 derivatives at the clorobiocin binding site via molecular docking. Molecular docking was accomplished for arylthiourea derivatives to comprehend their possible intermolecular interactions with the receptor. Clorobiocin is a based coumarin antibiotics, which prohibits the cell division of bacteria by inhibition of the DNA gyrase enzyme^51–54^.

**Figure 3:**
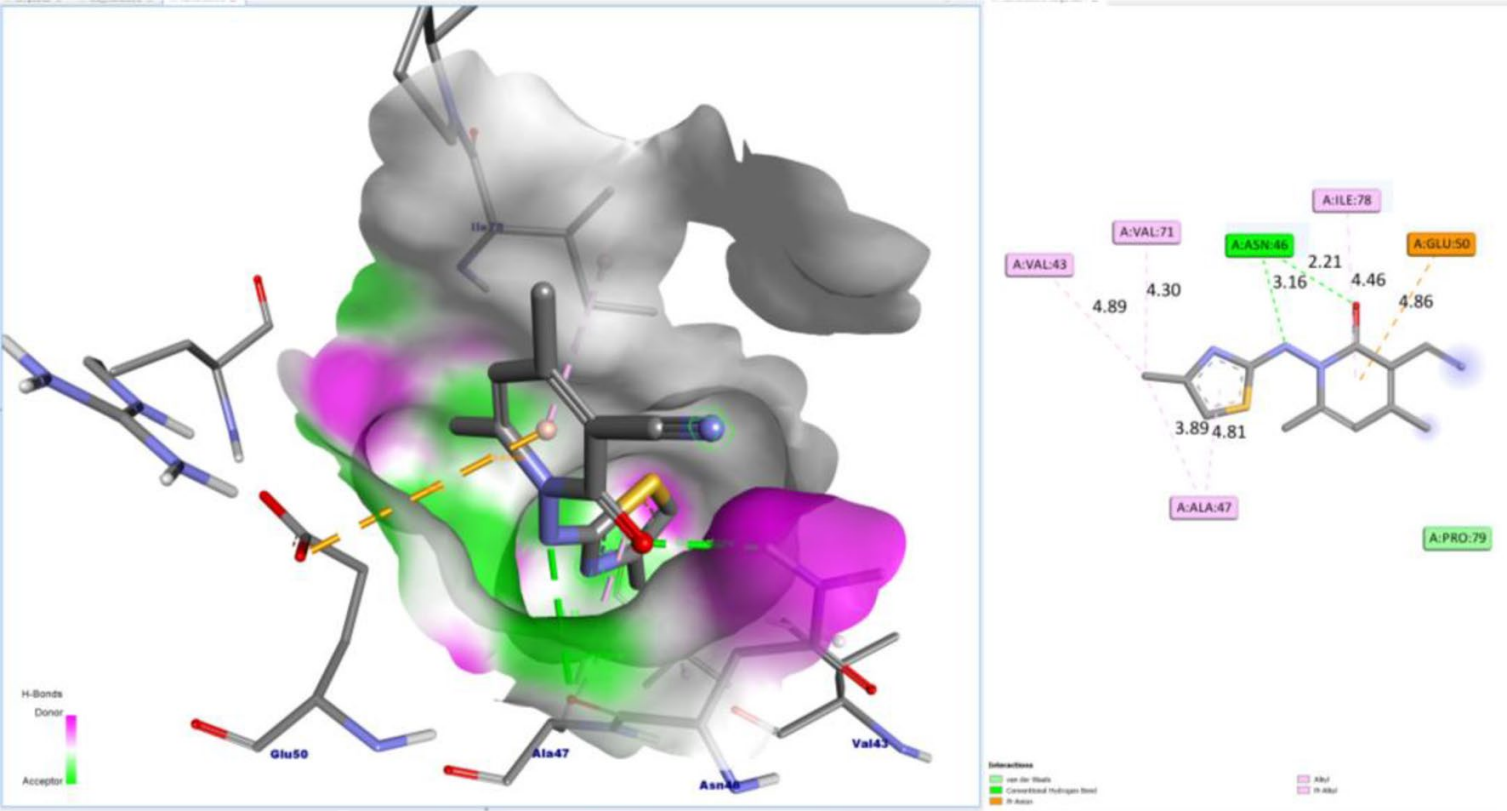
Docked conformation of compound **9** in the binding site of DNA-gyrase. Hydrogen bonds are shown by green dashed line and the other colors represent the hydrophobic interactions.

**Figure 4:**
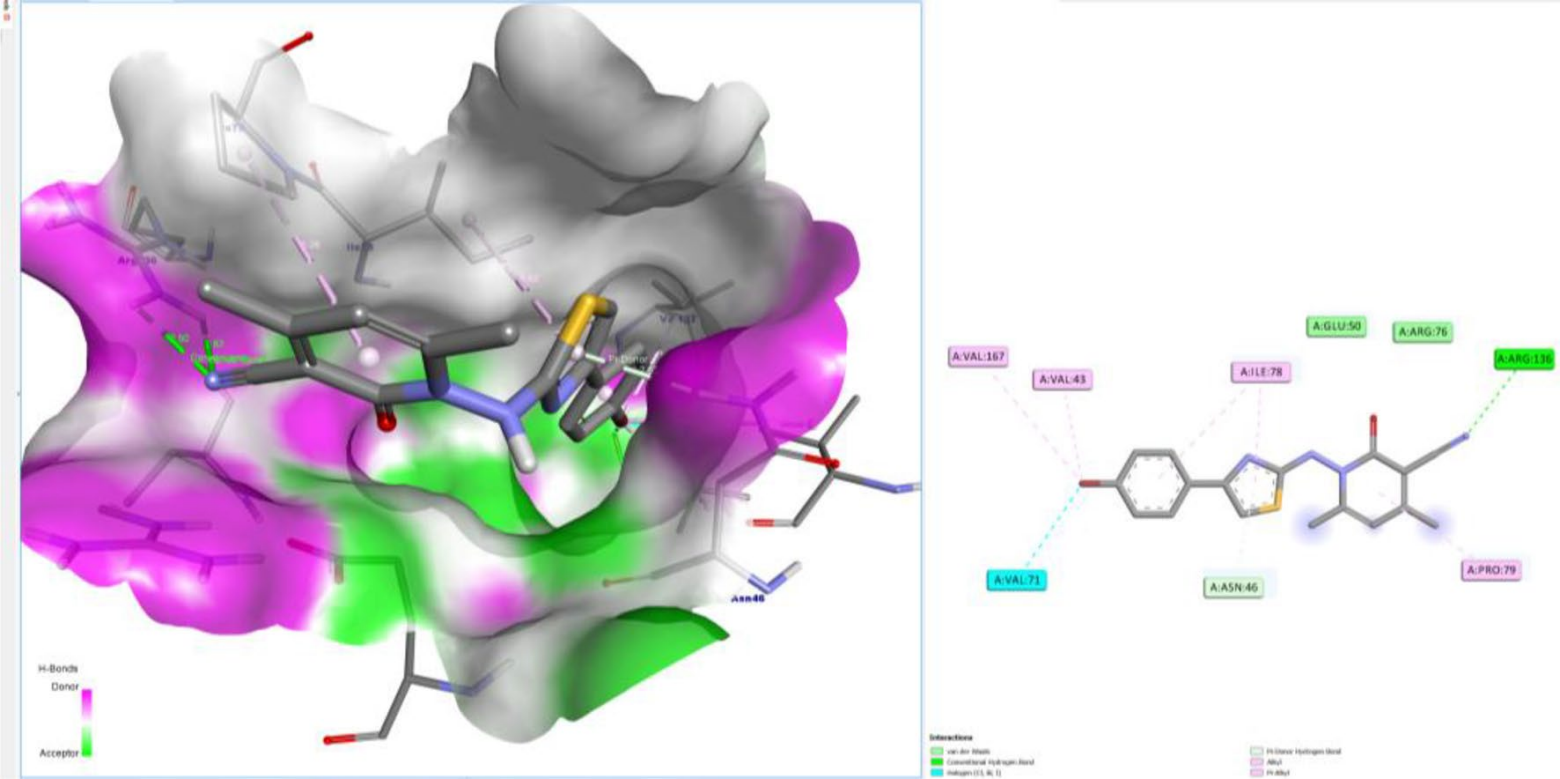
Docked conformation of compound **13a** in the binding site of DNA-gyrase. Hydrogen bonds are shown by green dashed line and the other colors represent the hydrophobic interactions.

Table 1 summarizes the binding depiction of the arylthioureas with DNA gyrase. The poses obtained from the docking procedure was selected due to their binding energy (~ − 6 – − 9 kcal/mol). Figures 3 and 4 showed 3D schematic interactions of compounds **13a** and **9** into the chlorobiocin binding site and showed that the compounds are fit to the binding pocket. These hydrophobic sites and hydrogen bond interactions of the derivatives are conserved in the majority of our compounds (Figs. 3 and 4).

**Table 1:** Energy-based interactions and hydrogen bonds of arylthiourea derivatives docked into DNA gyrase.

No	Estimated free energy of binding (kcal/mol)	Hydrogen bonds (distance)
2	− 6.4	Arg76 (2.92 A°),Gly77 (2.52 A°), Thr165 (2.39 A°), Asp73 (2.34 A°), Asn46 (2.75 A°)
6a	− 7.7	Arg76 (2.77 A°), Arg136 (1.95 A°)
6b	− 7.8	Arg76 (3.02 A°)
6c	− 7.7	Arg76 (2.93)
9	− 8.8	Asn46 (2.21 A°), Asn46 (3.16 A°)
13a	− 9.2	Arg136 (2.82 A°), Arg136 (2.5 A°)
13b	− 8.5	Ser121 (2.81 A°), His95 (3.03 A°), Ala96 (2.67 A°), Asn46 (2.14 A°)
15	− 7.6	Asn46 (2.6 A°), Asn (2.24 A°)
17	− 8.3	Asn46 (2.43 A°)

The docking results exhibited that some compounds (**9**, **13a** and **13b**) can produce a strong hydrophobic interaction and hydrogen bonds with Arg136 and Asn46 in the binding site. It is exciting that more complex stabilization could result from the hydrogen bonds between these compounds and Arg136 via cyano group in the pyridone ring (Figs. 3 and 4). Although these interactions were also observed for some other derivatives, but we think that the hydrophobic interaction is responsible for the activity variations.

Docked compounds also stabilize the DNA gyrase via hydrophobic interactions with Ala47, Glu50, Val71, Asp73, Arg76, Gly77, Ile78, Pro79, Met91, Val43, Thr165, and Val167. Compounds **9** and **13a** were pointedly embedded into the hydrophobic part of the pocket. All compounds showed that the hydrophobic pocket of the inhibitor pocket was occupied by pyridine, phenyl or substituted phenyl.

The docking method approved in this study was validated by redocking of chlorobiocin to the DNA gyrase protein. The residues Asp73, Asn46, and Arg136 are vital in making hydrogen bonds and are very important for the biological activity^55^ and in our study some compounds also displayed a strong hydrogen bond with Asn46. The highest dock score for our derivatives was − 9.2 and − 8.8 kcal/mol for compounds **13a** and **9**, respectively. The remainder molecules exhibited a docking scores ranging from − 8.5 to − 6.4 kcal/mol. Thus, the binding model stated here, proposes that arylthiourea derivatives act as DNA gyrase inhibitors and display some key structural points to be used in further optimization.

The biological assay (Tables 2 and 3), some compounds exhibited a strong activity against both the Gram-positive and Gram-negative bacterial. Gained results confirmed that compounds **9** had high activities against *E. coli* and *P. aeruginosa* with MIC 93.7 μg/mL. Also, compound **13a** showed the superlative activity against *E. coli*, *P. aeruginosa, S. aureus*, and *B. subtilis* with MIC 93.7, 62.5, 46.9, and 62.5 μg/mL, respectively. Also compound **13a** has shown the highest activity with MIC 7.8 and 5.8 μg/mL against *C. albicans and Aspergillus flavus,* respectively.

**Table 2:** In vitro antimicrobial activity of the synthesized compounds ^a,b^.

Diameter of inhibition zone (mm)						
Compounds	*E. coli*	*P. aeruginosa*	*S. aureus*	*B. subtilis*	*C. albicans*	*A. flavus*
1	NA	NA	NA	NA	2 ± 0.53	4 ± 0.66
2	10 ± 0.92	12 ± 1.02	16 ± 0.97	15 ± 1.36	16 ± 1.38	19 ± 1.60
6a	10 ± 0.87	13 ± 1.17	17 ± 1.06	15 ± 1.41	16 ± 1.52	21 ± 1.52
6b	13 ± 1.13	16 ± 1.48	20 ± 1.47	17 ± 1.61	19 ± 1.91	22 ± 1.53
6c	8 ± 0.79	12 ± 1.18	14 ± 1.35	13 ± 1.42	12 ± 1.18	16 ± 1.37
9	18 ± 0.94	17 ± 0.89	21 ± 1.73	18 ± 1.74	20 ± 1.67	23 ± 1.06
13a	21 ± 1.36	20 ± 1.45	22 ± 1.69	19 ± 1.68	20 ± 0.49	24 ± 1.75
13b	12 ± 1.24	15 ± 1.26	19 ± 1.53	16 ± 1.48	13 ± 1.25	17 ± 1.14
15	5 ± 0.53	9 ± 0.84	7 ± 0.82	5 ± 0.74	6 ± 0.80	7 ± 0.92
17	7 ± 0.86	10 ± 0.97	13 ± 1.18	12 ± 1.19	11 ± 1.46	15 ± 1.48
Ampicillin	25 ± 1.48	23 ± 1.28	24 ± 1.04	23 ± 0.93	–	–
Clotrimazole	–	–	–	–	27 ± 2.37	25 ± 1.91

^a^ Antimicrobial activity expressed as inhibition diameter zones in millimeters (mm) of synthesized compounds against the pathological strains based on well diffusion assay.

^b^ The experiment was carried out in triplicate and the average zone of inhibition was calculated.

^c^ NA No activity.

**Table 3:** Minimum inhibitory concentration (MIC) in (μg/mL) for compounds **2**, **6a**, **6b**, **9**, and **13a**^a^.

Compounds	*E. coli*	*P. aeruginosa*	*S. aureus*	*B. subtilis*	*C. albicans*	*A. flavus*
**2**	375 ± 3.00	250 ± 2.25	187.5 ± 8.08	250 ± 1.00	62.5 ± 0.50	31.2 ± 0.62
**6a**	250 ± 3.21	187.5 ± 8.23	125 ± 0.00	187.5 ± 0.50	46.9 ± 0.84	23.4 ± 0.50
**6b**	187.5 ± 0.50	125 ± 1.73	125 ± 1.00	187.5 ± 0.58	31.2 ± 0.62	11.7 ± 0.30
**9**	93.7 ± 0.95	93.7 ± 0.95	125 ± 0.58	187.5 ± 0.50	23.4 ± 0.84	15.6 ± 0.76
**13a**	93.7 ± 0.95	62.5 ± 2.00	46.9 ± 0.84	62.5 ± 0.50	7.8 ± 0.17	5.8 ± 0.65
Ampicillin	125 ± 0.58	125 ± 3.51	187.5 ± 0.06	125 ± 1.73	–	–
Clotrimazole	–	–	–	–	5.8 ± 0.06	3.9 ± 0.06

^a^The experiment was carried out in triplicate and the average was calculated.

The observed results displayed that compound **13a** has better biological results than other arylthioureas. Existence of electron-withdrawing group (bromine) at *p*-position of the phenyl ring could be accountable for good activities due to its size and inductive effect.

## Experiment

### General

Melting points were recorded on digital Gallen-Kamp MFB-595 apparatus and are uncorrected. IR spectra were recorded on Schimadzu FTIR 440 spectrometer using KBr pellets. Mass spectra were performed at 70 eV on an MS-50 Kratos (A.E.I.) spectrometer provided with a data system. ^1^H NMR (500 MHz) and ^13^C NMR (125 MHz) spectra were recorded on a Bruker model Ultra Shield NMR spectrometer using CDCl_3_ or DMSO-d_6_ with TMS as an internal standard. Chemical shifts are reported as δ ppm units. The monitoring of the progress of reactions and homogeneity of the products was carried out using thin layer chromatography (TLC).

*1-(3-Cyano-4,6-dimethyl-2-oxopyridin-1(2H)-yl)thiourea* (**2**). 1-Amino-4,6-dimethyl-2-oxo-1,2-dihydropyridine-3-carbonitrile (16.3 g, 0.1 mol) was dissolved in coc. HCl (40 mL) and ammonium isothiocynte was added (7.6 g, 0.1 mol). The mixture was reflux for 1 h. After cooling, the white precipitate was filtered off, washed with ethanol, and dried under reduced pressure. White crystals, yield (95%), mp 249–250° C. IR (KBr) ν (cm^−1^): 3408 (NH), 3261, 3219 (NH_2_), 2222 (CN), 1662 (C=O), 1624 (C=C), 1269 (C=S); ^1^H NMR (500 MHz, CDCl_3_) δ (ppm): 2.20 (s, 3H, CH_3_), 2.27 (s, 3H, CH_3_), 6.32 (s, 1H, pyridine-H_5_), 7.76 (s, D_2_O exchangeable, 2H, NH_2_), 10.16 (s, D_2_O exchangeable, H, NH); ^13^C NMR (125 MHz, CDCl_3_) *δ*_C_ (ppm): 18.6 (CH_3_), 20.7 (CH_3_), 101, 108.9, 115.6 (CN), 153.8, 155.5, 159.9 (C=O), 185.5 (C=S); MS *m/z* (%): 223.6 [M^+^ + 1] (5%), 204.9 [M^+^-H_2_O], 163, 148, 119 (100); Anal. Calcd. for C_9_H_10_N_4_OS (222.27): C, 48.63; H, 4.54; N, 25.21, Found: C, 48.43; H, 4.36; N, 25.03%.

### General procedure for synthesis thiazole derivatives 6, 9, 13, 15, and 17^56^

Equimolar amounts of **2** (1 mmol) and 2-oxo-*N*-arylpropanehydrazonoyl chloride **3a**-**c**; chloroacetone; *α-*bromoketones **11a,b**; ethyl chloroacetate; and 2,3-dichloroquinoxaline (1 mmol) in absolute ethanol (30 mL) {few drops of triethylamine was added in case of **3a-c** and chloroacetone} was heated under reflux for 3–6 h (TLC), then left to cool. The solid was isolated by filtration, washed with ethanol, dried, and recrystallized from (EtOH).

*4,6-Dimethyl-1-((4-methyl-5-(phenyldiazenyl)thiazol-2-yl)amino)-2-oxo-1,2-dihydropyridine-3-carbonitrile* (**6a**). Orange crystals, yield (86%), mp 234–235 °C (EtOH); IR (*ν*_max_, cm^−1^): 3219w (NH), 2218 s (CN), 1643 s (C=O), 1578-1485 s (C=C); ^1^H NMR (500 MHz, CDCl_3_) *δ*_H_ (ppm): 2.25 (s, 3H, CH_3_), 2.35 (s, 3H, CH_3_), 2.36 (s, 3H, CH_3_), 6.33 (s, 1H, pyridine-H_5_), 7.33 (t, 2H, Ar–H), 7.44 (t, 2H, Ar–H), 7.52 (d, 1H, *J* = 8.5 Hz, Ar–H), 9.88 (s, D_2_O exchangeable, 1H, NH); ^13^C NMR (125 MHz, DMSO) *δ*_C_ (ppm): 18.8 (CH_3_), 20.7 (CH_3_), 21.5 (CH_3_), 108, 114, 117, 128.6, 128.8, 129.2, 129.4, 135.7, 138, 147, 154, 160, 167; MS *m/z* (%): 364 [M^+^] (3%), 252 (15), 163 (55), 126 (100); Anal. Calcd. for C_18_H_16_N_6_OS (364.43): C, 59.33; H, 4.43; N, 23.06, Found: C, 59.07; H, 4.19; N, 22.89%.

*4,6-Dimethyl-1-((4-methyl-5-(p-tolyldiazenyl)thiazol-2-yl)amino)-2-oxo-1,2-dihydropyridine-3-carbonitrile* (**6b**). Orange crystals, yield (85%), mp 245–246 °C (EtOH); IR (*ν*_max_, cm^−1^): 3219w (NH), 2222 s (CN), 1656 s (C=O), 1575–1490 s (C=C); ^1^H NMR (500 MHz, CDCl_3_) *δ*_H_ (ppm): 2.26 (s, 3H, CH_3_), 2.33 (s, 3H, CH_3_), 2.37 (s, 3H, CH_3_), 2.49 (s, 3H, CH_3_), 6.31 (s, 1H, pyridine-H_5_), 7.20 (d, 2H, *J* = 6.5 Hz, Ar–H), 7.55 (d, 2H, *J* = 6.5 Hz, Ar–H), 10.08 (s, D_2_O exchangeable, 1H, NH); ^13^C NMR (125 MHz, DMSO) *δ*_C_ (ppm): 19 (CH_3_), 20.7 (CH_3_), 20.8 (CH_3_), 20.9 (CH_3_), 100, 108.6, 109, 115, 116, 116.4, 129.4, 135.3, 147, 150.2, 154, 160, 167; MS *m/z* (%): 363 [M^+^-15] (4%), 232 (60), 163 (45), 120 (100); Anal. Calcd. for C_19_H_18_N_6_OS (378.45): C, 60.30; H, 4.79; N, 22.21, Found: C, 60.30; H, 4.79; N, 22.21%.

*1-((5-((4-Chlorophenyl)diazenyl)-4-methylthiazol-2-yl)amino)-4,6-dimethyl-2-oxo-1,2-dihydropyridine-3-carbonitrile* (**6c**). Yellow crystals, yield (85%), mp 238–239 °C (EtOH); IR (*ν*_max_, cm^−1^): 3217w (NH), 2222 s (CN), 1653 s (C=O), 1585–1489 s (C=C); ^1^H NMR (500 MHz, CDCl_3_) *δ*_H_ (ppm): 2.24 (s, 3H, CH_3_), 2.31 (s, 3H, CH_3_), 2.49 (s, 3H, CH_3_), 6.34 (s, 1H, pyridine-H_5_), 7.33 (d, 2H, J = 6.5 Hz, Ar–H), 7.44 (d, 2H, *J* = 6.5 Hz, Ar–H), 11 (s, D_2_O exchangeable, 1H, NH); ^13^C NMR (125 MHz, DMSO) *δ*_C_ (ppm): 16.83 (CH_3_), 18.7 (CH_3_), 20.6 (CH_3_), 100, 108, 109, 115, 116, 116.4, 129.4, 135.3, 147, 150.2, 154, 160, 167; MS *m/z* (%): 384 [M^+^-15] (5%), 252 (15), 163 (55), 126 (100); Anal. Calcd. for C_18_H_15_ClN_6_OS (398.87): C, 54.20; H, 3.79; N, 21.07, Found: C, 53.83; H, 3.61; N, 20.87%.

*4,6-Dimethyl-1-((4-methylthiazol-2-yl)amino)-2-oxo-1,2-dihydropyridine-3-carbonitrile* (**9**). White crystals, yield (85%), mp 219–220 °C (EtOH); IR (*ν*_max_, cm^−1^): 3261w (NH), 2216 s (CN), 1654 s (C=O), 1575–1543 s (C=C); ^1^H NMR (500 MHz, CDCl_3_) *δ*_H_ (ppm): 2.07 (s, 3H, CH_3_), 2.31 (s, 3H, CH_3_), 2.49 (s, 3H, CH_3_), 6.34 (s, 1H, pyridine-H_5_), 6.42 (s, 1H, thiazole-H_5_), 10.77 (s, D_2_O exchangeable, 1H, NH); ^13^C NMR (125 MHz, DMSO) *δ*_C_ (ppm): 16.6 (CH_3_), 19.4 (CH_3_), 21.1 (CH_3_), 100, 101.4, 109, 116, 154.9, 156, 159.4, 181.7, 185.8; MS *m/z* (%): 260 [M^+^] (100%), 243 (15), 148 (52); Anal. Calcd. for C_12_H_12_N_4_OS (260.32): C, 55.37; H, 4.65; N, 21.52, Found: C, 55.15; H, 4.32; N, 21.11%.

## Method 2

### Synthesis of compound 6b from compound 9^57^

To a stirred solution of compound **9** (0.5206 g, 2 mmol) in ethanol (30 mL) sodium acetate trihydrate (0.26 g, 2 mmol) was added. After stirring for 15 min, the mixture was chilled at 0 °C and treated with a cold solution of *p*-toluidine (0.2 g, 2 mmol) in 6 M hydrochloric acid (1.5 mL) with sodium nitrite solution (0.14 g, 2 mmol) in water (3 mL). The addition of the diazonium salt was stirred for an additional 2 h at 0–5 °C and then left for 8 h in a refrigerator (4 °C). The resulting solid was collected by filtration, washed thoroughly with water and dried. The crude product was crystallized from ethanol.

*1-((4-(4-Bromophenyl)thiazol-2-yl)amino)-4,6-dimethyl-2-oxo-1,2-dihydropyridine-3-carbonitrile* (**13a**). White crystals, yield (85%), mp 233–235 °C (EtOH); IR (*ν*_max_, cm^−1^): 3261w (NH), 2222 s (CN), 1662 s (C=O), 1593–1537 s (C=C); ^1^H NMR (500 MHz, CDCl_3_) *δ*_H_ (ppm): 2.33 (s, 3H, CH_3_), 2.49 (s, 3H, CH_3_), 6.47 (s, 1H, pyridine-H_5_), 7.53 (s, 1H, thiazole-H_5_), 7.56 (dd, 2H, *J* = 2 Hz, 9 Hz, Ar–H), 7.67 (dd, 2H, *J* = 2 Hz, 9 Hz, Ar–H), 10.81 (s, D_2_O exchangeable, 1H, NH); ^13^C NMR (125 MHz, DMSO) *δ*_C_ (ppm): 19 (CH_3_), 20.8 (CH_3_), 100, 106.9, 108.5, 115.6, 120.9, 127.6, 131.6, 133.1, 148.8, 153.8, 158.8, 160, 167.5; MS *m/z* (%): 401 [M^+^  + 1] (100%), 256 (70); Anal. Calcd. for C_17_H_13_BrN_4_OS (401.28): C, 50.88; H, 3.27; N, 13.96, Found: C, 50.49; H, 3.11; N, 13.71%.

*4,6-Dimethyl-2-oxo-1-((4-(2-oxo-2H-chromen-3-yl)thiazol-2-yl)amino)-1,2-dihydropyridine-3-carbonitrile* (**13b**). White crystals, yield (85%), mp 280–281 °C (EtOH); IR (*ν*_max_, cm^−1^): 3170w (NH), 2223 s (CN), 1739, 1724 (C=O), 1662 s (C=O), 1583–1531 s (C=C); ^1^H NMR (500 MHz, CDCl_3_) *δ*_H_ (ppm): 2.41 (s, 3H, CH_3_), 2.49 (s, 3H, CH_3_), 6.50 (s, 1H, pyridine-H_5_), 7.35 (ddd, 1H, *J* = 1.5 Hz, 8.5 Hz, coumarin-H_6_), 7.42 (d, 1H, *J* = 8.5 Hz, coumarin-H_8_), 7.60 (ddd, 1H, *J* = 1.5 Hz, 8.5 Hz, coumarin-H_7_), 7.84 (dd, 1H, *J* = 1.5 Hz, 9.5 Hz, coumarin-H_5_), 8.36 (s, 1H, coumarin-H_4_), 10.85 (s, D_2_O exchangeable, 1H, NH); ^13^C NMR (125 MHz, DMSO) *δ*_C_ (ppm): 19 (CH_3_), 20.9 (CH_3_), 100.2, 108.7, 112.5, 115.4, 115.9, 119, 120.1, 124.7, 129, 131.9, 138.7, 143.5, 152.3, 155, 158.6, 158.8 (CO), 160.2 (CO, lactone), 167.2; MS *m/z* (%): 390 [M^+^] (8%), 244 (70), 148 (100), 119 (60); Anal. Calcd. for C_20_H_14_N_4_O_3_S (390.42): C, 61.53; H, 3.61; N, 14.35, Found: C, 61.28; H, 3.35; N, 14.21%.

*4,6-Dimethyl-2-oxo-1-((4-oxo-4,5-dihydrothiazol-2-yl)amino)-1,2-dihydropyridine-3-carbonitrile* (**15**). White crystals, yield (85%), mp 233–235 °C (EtOH); IR (*ν*_max_, cm^−1^): 3250w (NH), 2216 s (CN), 1737s (C=O), 1699 (C=O); ^1^H NMR (500 MHz, CDCl_3_) *δ*_H_ (ppm): 2.23 (s, 3H, CH_3_), 2.33 (s, 3H, CH_3_), 4 (s, 2H, CH_2_, thiazole), 6.39 (s, 1H, pyridine-H_5_), 12.53 (s, D_2_O exchangeable, 1H, NH); ^13^C NMR (125 MHz, DMSO) *δ*_C_ (ppm): 18.8 (CH_3_), 20.51 (CH_3_), 34.4 (CH_2_), 99.6, 108.1, 115.8, 151, 155.9, 157.5, 171.4 (CO), 173.7 (CO); MS *m/z* (%): 262 [M^+^] (90%), 215 (100), 148 (30); Anal. Calcd. for C_11_H_10_N_4_O_2_S (262.29): C, 50.19; H, 3.53; N, 21.15, Found: C, 50.37; H, 3.84; N, 21.36%.

*4,6-Dimethyl-2-oxo-1-(thiazolo[4,5-b]quinoxalin-2-ylamino)-1,2-dihydropyridine-3-carbonitrile* (**17**). Brown powder, yield (85%), mp ˃300 °C (EtOH); IR (*ν*_max_, cm^−1^): 3151w (NH), 2224 s (CN), 1684 (C=O); ^1^H NMR (500 MHz, CDCl_3_) *δ*_H_ (ppm): 2.32 (s, 3H, CH_3_), 2.33 (s, 3H, CH_3_), 6.34 (s, 1H, pyridine-H_5_), 7.07–7.10 (m, 2H, Ar–H); 7.89–7.93 (m, 2H, Ar–H); 10.17 (s, D_2_O exchangeable, 1H, NH); ^13^C NMR (125 MHz, DMSO) *δ*_C_ (ppm): 18.5 (CH_3_), 19.2 (CH_3_), 108.9, 115.1 (CN), 123, 125.6, 128.3, 132, 140, 153.8, 155.5, 158.6, 160, 181.4 (CO); MS *m/z* (%): 323 [M^+^-CN] (50%), 322 (100); Anal. Calcd. for C_17_H_12_N_6_OS (348.38): C, 58.61; H, 3.47; N, 24.12, Found: C, 58.23; H, 3.19; N, 23.94%.

### Molecular docking studies

The structure of our target enzyme (PDB code 1KZN) was chosen as the protein model for this study^58^. The heteroatoms were taken away from the protein file and the resulting structure was introduced to AutoDock. The binding image of 9 new arylthioureas with DNA gyrase were assessed in the same way of binding of clorobiocin.

The 3D structures of arylthioureas were optimized using GAMESS (https://www.msg.chem.iastate.edu/gamess). The final forms were calculated with the semi empirical parameterized model number 3 (PM3) method.

Docking was executed by the default parameters of molecular docking AutoDock 4.2 and employed empirical free energy function^59^. In the docking procedure, compounds were supposed to be flexible and the docking software was allowed to rotate all rotatable bonds of them to obtain the best conformer within the active site of the enzyme. Clorobiocin was redocked to the binding site to evaluate our method.

The grid box was positioned with the coordinates x = 19.172, y = 30.465, z = 34.697 for DNA gyrase (PDB code 1KZN). Grid box sizes were 60 × 60 × 60 with a 0.5 Å grid points space. Grid maps were calculated by Autogrid4. A lamarckian genetic algorithm within the Autodock was used to estimate the diverse ligand conformers. Conformations were clustered by the root mean square deviation tolerance of 2.0 Å and were ranked according to the binding free energy^59^. Discovery Studio 2020 Visualizer was used to explore the hydrophobic and hydrogen bonding interactions of the compound with DNA gyrase.

### Antimicrobial evaluation

The agar well diffusion method is widely used to evaluate the antimicrobial activity of plants or microbial extracts. Similar to the procedure used in disk-diffusion method, the agar plate surface is inoculated by spreading a volume of the microbial inoculum over the entire agar surface then, a hole with a diameter of 6 to 8 mm is punched aseptically with a sterile corkborerora tip A volume (20–100 mL) of the antimicrobial agent or extract solution at desired concentration is introduced into the well and agar plates are then incubated under suitable conditions depending upon the microorganism. The antimicrobial agent diffusion the agar medium and inhibits the growth of the microbial strain^60^.
